# Genome-Wide Analysis of Phosphorus Transporter Genes in *Brassica* and Their Roles in Heavy Metal Stress Tolerance

**DOI:** 10.3390/ijms21062209

**Published:** 2020-03-23

**Authors:** Yuanyuan Wan, Zhen Wang, Jichun Xia, Shulin Shen, Mingwei Guan, Meichen Zhu, Cailin Qiao, Fujun Sun, Ying Liang, Jiana Li, Kun Lu, Cunmin Qu

**Affiliations:** 1 Chongqing Engineering Research Center for Rapeseed, College of Agronomy and Biotechnology, Southwest University, No. 2 Tiansheng Road, Beibei, Chongqing 400715, China; wyywinyuan@swu.edu.cn (Y.W.); wangzhen.cq@gmail.com (Z.W.); xjc199802@swu.edu.cn (J.X.); ssl9942@email.swu.edu.cn (S.S.); ghhooo1770@email.swu.edu.cn (M.G.); zmc0809@email.swu.edu.cn (M.Z.); qcl123@email.swu.edu.cn (C.Q.); drsunfujun@email.swu.edu.cn (F.S.); yliang@swu.edu.cn (Y.L.); ljn1950@swu.edu.cn (J.L.); 2 Academy of Agricultural Sciences, Southwest University, Chongqing 400715, China

**Keywords:** *Brassica* species, *Brassica napus* L., phosphorus transporter (PHT), evolution, expression profiles, heavy metal

## Abstract

Phosphorus transporter (*PHT*) genes encode H_2_PO^4−^/H^+^ co-transporters that absorb and transport inorganic nutrient elements required for plant development and growth and protect plants from heavy metal stress. However, little is known about the roles of *PHTs* in *Brassica* compared to *Arabidopsis thaliana*. In this study, we identified and extensively analyzed 336 PHTs from three diploid (*B. rapa, B. oleracea,* and *B. nigra*) and two allotetraploid (*B. juncea* and *B. napus*) *Brassica* species. We categorized the *PHTs* into five phylogenetic clusters (*PHT1*–*PHT5*), including 201 *PHT1* homologs, 15 *PHT2* homologs, 40 *PHT3* homologs, 54 *PHT4* homologs, and 26 *PHT5* homologs, which are unevenly distributed on the corresponding chromosomes of the five *Brassica* species. All *PHT* family genes from *Brassica* are more closely related to Arabidopsis *PHTs* in the same vs. other clusters, suggesting they are highly conserved and have similar functions. Duplication and synteny analysis revealed that segmental and tandem duplications led to the expansion of the *PHT* gene family during the process of polyploidization and that members of this family have undergone purifying selection during evolution based on Ka/Ks values. Finally, we explored the expression profiles of *BnaPHT* family genes in specific tissues, at various developmental stages, and under heavy metal stress via RNA-seq analysis and qRT-PCR. *BnaPHTs* that were induced by heavy metal treatment might mediate the response of rapeseed to this important stress. This study represents the first genome-wide analysis of *PHT* family genes in *Brassica* species. Our findings improve our understanding of *PHT* family genes and provide a basis for further studies of *BnaPHTs* in plant tolerance to heavy metal stress.

## 1. Introduction

Phosphorus (Pi), one of the three major mineral nutrients required for plant growth and development, plays an important role in the plant lifecycle [1]. Phosphorus transporter (*PHT*) genes encode H_2_PO^4−^/H^+^ co-transporters that are responsible for the absorption and transport of soil-available phosphorus in plant roots, which are divided into five subfamilies: *PHT1*, *PHT2*, *PHT3*, *PHT4*, and *PHT5* [2]. Among these, *PHT1 (plasma membrane)* genes have been well characterized in a wide range of species, such as *Arabidopsis thaliana* [3], *B*. *napus* [4,5], graminaceous species [6,7,8,9,10], solanaceous species [11,12], and legumes [13,14,15,16]. The main function of *PHT1* transporters is to mediate the absorption and transport of Pi from the soil [17]. Most *PHT1* family genes are expressed exclusively or predominantly in both roots and shoots, which are strongly induced by Pi starvation or by inoculation with arbuscular mycorrhiza [3,6,9,10,15,18]. 

In general, *PHT1s* function in Pi acquisition from the soil, whereas *PHT2* (plastid inner envelope), *PHT3* (mitochondrial inner membrane), *PHT4* (mostly plastid envelope and one Golgi-localized transporter), and *PHT5* (vacuole membrane) family genes are mainly responsible for maintaining the Pi distribution within the plant [19] and have thus far received less attention than *PHT1* family genes [20]. For example, the chloroplast envelope-localized gene *PHT2* is primarily expressed in green tissues and encodes a protein thought to function in Pi transport into leaves and tolerance to Pi starvation [1,21,22,23]. The first putative mitochondrial Pi transporter genes identified [24] were *PHT3* genes, which are highly conserved within the mitochondrial transporter family [2] and are involved in Pi exchange between the mitochondrial matrix and cytosol [25]. *PHT4* family genes also function in multiple biological processes, as they not only play important roles in Pi transport in plastids and the Golgi apparatus [26,27], but they might also be involved in plant growth [28], carbon metabolism [29,30], pathogen resistance [31,32], and salt tolerance [33]. *PHT3* and *PHT4* family members might also contribute to Pi transport within the plant tissues under Pi starvation [27,34]. PHT5 family proteins, also known as SYG1, PHO81, and XPR1 (SPX)-Major Facility Superfamily (MFS) proteins, are located in the vacuole membrane and are involved in maintaining Pi homeostasis within the plant [35,36,37]. These studies focused on the roles of PHT family proteins in Pi uptake from the soil and distribution within plants [1,19,20].

In nature, many heavy metals serve as essential trace elements for plant growth and development, including copper, manganese, cobalt, zinc, and chromium [38]. However, when the content of a heavy metal in the environment exceeds a certain critical value, it can have toxic effects on plants, disrupting plant metabolic processes, inhibiting plant growth or causing plant death [39,40]. Many species or genotypes of *Brassica* have a strong ability to absorb and enrich heavy metals, making them ideal for the phytoremediation of heavy metal-contaminated soils, particularly *B. juncea* and *B. napus* [39,41,42,43,44,45]. The uptake of Pi and arsenic depends on the same transport system in plants [46,47,48]. *PHT1* family genes function in the acquisition of As and Pi, such as *PHT1;1* and *PHT1;4* in Arabidopsis [49,50], *OsPHT1;8* in rice [51], and *PvPHT1;3* in *Pteris vittata* [46]. However, a comprehensive analysis of the entire *PHT* family (*PHT1, PHT2, PHT3, PHT4,* and *PHT5*) in *Brassica* species has not been reported, and the mechanisms that contribute to the tolerance of *Brassica* plants to heavy metals are unclear.

*B. rapa* (AA, 2n = 20), *B. nigra* (BB, 2n = 16), and *B. oleracea* (CC, 2n = 18) contain three diploid genomes. Interspecific hybridization of these plants led to the formation of three amphidiploid plants, *B. juncea* (AABB, 2n = 36), *B. napus* (AACC, 2n = 38), and *B. carinata* (BBCC, 2n = 34), and their relationships among these plants are described by the U’s triangle model [52], providing an excellent evolutionary model for investigating the expansion of gene families [53]. In the present study, using genome-wide analysis, we comprehensively identified the phosphorus transporter gene family in five *Brassica* species (*B. rapa*, *B. nigra*, *B. oleracea*, *B. juncea*, and *B. napus*), including the *PHT1, PHT2, PHT3, PHT4*, and *PHT5* gene families. We analyzed their duplication and classification, chromosome distribution, and motifs and performed a phylogenetic analysis. Finally, we investigated the expression patterns of all *BnaPHT* genes in various tissues and the expression profiles of various genes in *B*. *napus* in response to cadmium and arsenic treatment. Our results provide a foundation for functional genomic studies of the *PHT* gene family in *Brassica*.

## 2. Results

### 2.1. Identification and Evolutionary Relationships of PHT Family Genes

**Figure 1:**
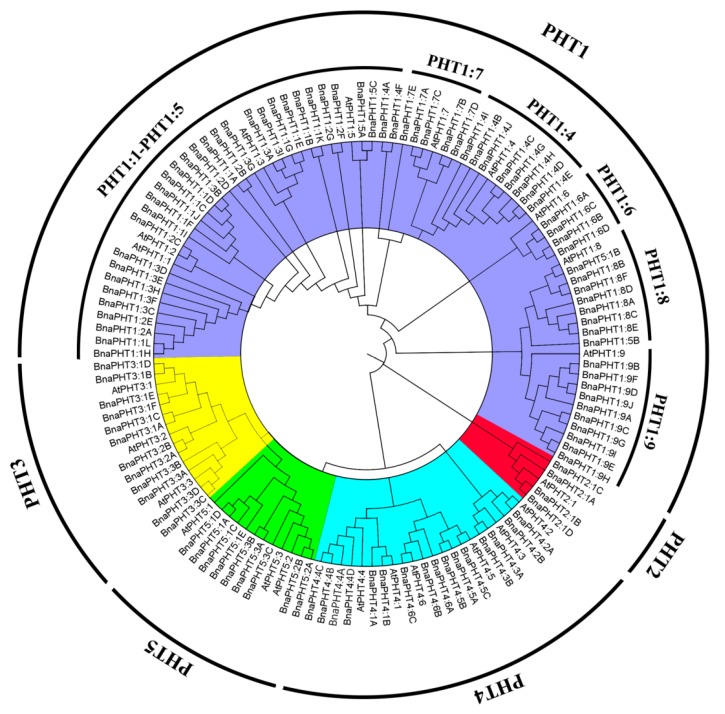
Phylogenetic tree of PHT proteins from *A. thaliana* and five *Brassica* species. The phylogenetic tree was constructed using the neighbor-joining method with 1000 bootstrap replicates in MEGA7 (https://www.megasoftware.net/) and visualized using FigTree v1.4.4 (http://tree.bio.ed.ac.uk/software/figtree/). The *PHTs* were divided into five subfamilies (*PHT1–PHT5*), which are indicated by different colors. Gene names and accession numbers are shown in Table S1.

We identified 336 *PHT* genes in the genomes of five *Brassica* plants (54 from *B. rapa,* 34 from *B. oleracea,* 55 from *B. nigra,* 85 from *B. juncea,* and 108 from *B. napus*) using the protein sequences of 22 *PHT* family genes as queries in the The Arabidopsis Information Resource (TAIR10) database (Table 1 and Table S1). We aligned the deduced amino acid sequences of all 108 PHT proteins with those of *A. thaliana* and performed phylogenetic analysis. The phylogenetic tree was divided into five subgroups, *PHT1* to *PHT5* (Figure 1 and Table S1). All PHT proteins in *Brassica* were classified into known subfamilies with *A. thaliana*, suggesting that they diverged from a common ancestor. Of the five subgroups identified, subgroup *PHT1* contained the largest number of *PHT* genes (201 *PHT1s*), representing 59.82% of the total number of *PHT* genes among the five *Brassica* species. The encoded proteins ranged from 57 amino acids (*BnaPHT1:2E*) to 1958 amino acids long (*BjuPHT1:1A*). All *PHT1* subfamily members were divided into nine subgroups (*PHT1:1, PHT1:2, PHT1:3, PHT1:4, PHT1:5, PHT1:6, PHT1:7, PHT1:8,* and *PHT1:9*), including 24, 23, 23, 31, 8, 17, 15, 19, and 41 *PHT1* family members. *PHT1:1*, *PHT1:2,* and *PHT1:3* were grouped in the same subcluster (Table 1 and Table S1), suggesting they share a closer relationship than the others.

Additionally, we analyzed the physical and chemical properties of all *PHT* family genes and encoded proteins, including their chromosome locations, molecular weights (MWs), and theoretical Isoelectric points (pIs) (Table S1). For example, the 15 genes in the *PHT2* subfamily encode deduced proteins ranging from 433 (*BolPHT2:1C*) to 760 (*BjuPHT2:1B*) amino acids long, representing the smallest subgroups. The 40 *PHT3* subfamily genes encode 211 (*BnaPHT3:3C*) to 849 (*BjuPHT3:2A*) amino acid proteins, the 54 *PHT4* subfamily genes encode 102 (*BjuPHT4:2A*) to 1318 (*BniPHT4:3A*) amino acid proteins, and the 26 *PHT5* subfamily genes encode 138 (*BnaPHT5:1B*) to 1056 (*BolPHT5:3A*) amino acid proteins (Table S1). These results suggest that this protein family might have lost partial amino acid sequences in *Brassica* during the process of polyploidization [54]. We, therefore, comprehensively investigated the expansion mechanisms and functional characteristics of *PHT* family genes in *A. thaliana* and five *B*rassica species. 

### 2.2. Chromosome Localizations of PHT Genes in the Five Brassica Species

**Figure 2:**
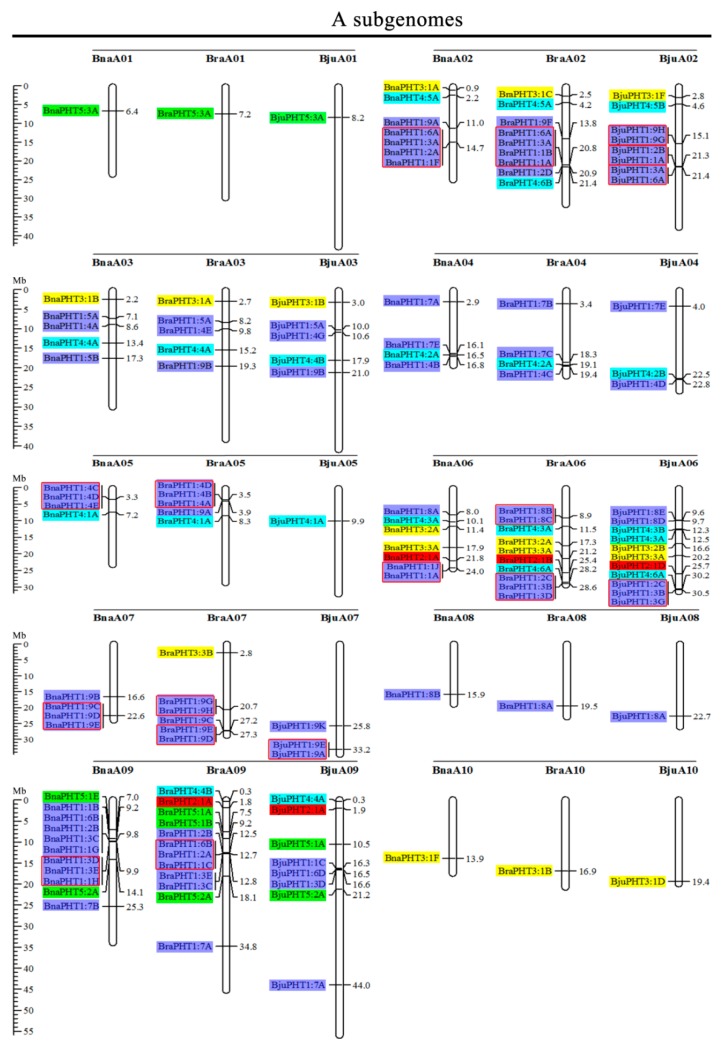

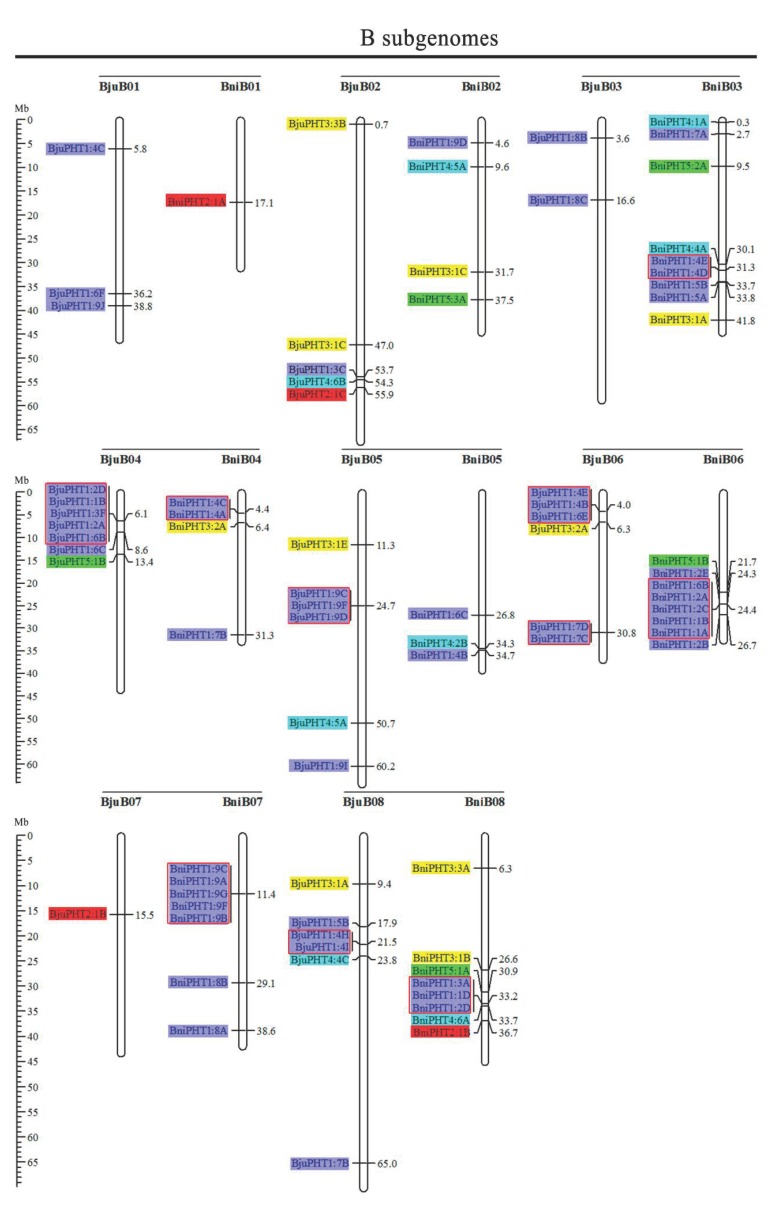

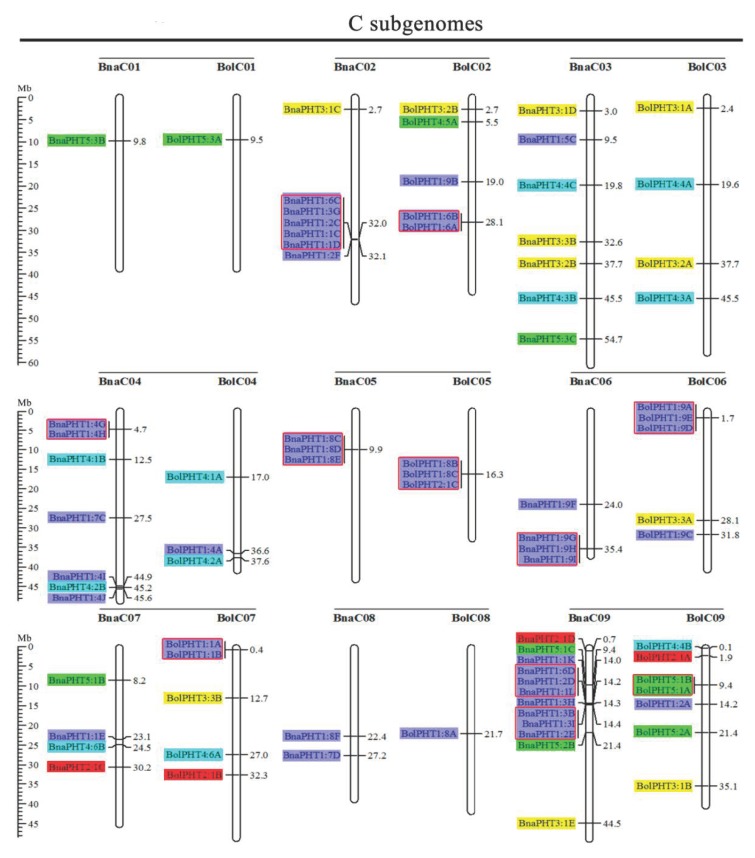
Chromosomal distribution and analysis of duplication events in *PHT* family genes among *Brassica* species. Genes from the same subtribe are indicated by the same color, which matches the color used for the corresponding family in the evolutionary tree. The labels on the corresponding chromosomes indicate the names of the source organism and the subgenome. The scales indicate the sizes of various *Brassica* plant genomes. Any two or more adjacent homologous genes on the same chromosome less than 100 kb apart are highlighted by red boxes. *Bra, B. rapa; Bol, B. oleracea; Bni, B. nigra; Bna, B. napus*; and *Bju, B. juncea.*

Based on the physical positions of the *PHT* genes annotated in the general feature format GFF files in the Brassica Database (BRAD), we marked the physical locations of 336 *Brassica PHT* genes on the physical maps of *B. rapa, B. oleracea, B. nigra, B. juncea*, and *B. napus* (Figure 2). In the *B*. *rapa* genome, 12 *PHT* genes are located on chromosome BraA09, which contains the most *PHT* genes, whereas chromosomes BraA01, BraA08, and BraA10 each contain only one *PHT* gene. In the *B. oleracea* genome, chromosome BolC09 contains the most *PHT* genes (7), whereas chromosomes BolC01 and BolC08 each contain only one *PHT* gene. In the *B. nigra* genome, chromosome BniB09 contains the most *PHT* genes (9), whereas chromosome BniB01 contains only one *PHT* gene. In the *B. juncea* genome, chromosome BjuA06 contains the most *PHT* genes (11), whereas chromosomes BjuA01, BjuA08, BjuA10, and BjuB07 each contain only one *PHT* gene. Finally, in the *B*. *napus* genome, 12 *PHT* genes are located on chromosome BnaC09, which contains the largest number of *PHT* genes, whereas chromosomes BnaA01, BnaA08, BnaA10, and BnaC01 each contain only one *PHT* gene. A total of 302 *PHT* family members were accurately mapped onto the 69 (BraA, BolC, BniB, BjuA, BjuB, BnaA, and BnaC) chromosomes (Figure 2), but the others (34 genes) were located in the 17 scaffold fragments (Table S1), and which were unevenly distributed on all the whole chromosomes, and generally more abundant at the both ends of the chromosomes in the five *Brassica* species (Figure 2).

### 2.3. Analysis of Duplication and Synteny of PHT Genes Among the Five Brassica Species

Segmental and tandem duplications play important roles in the occurrence of new gene functions and the amplification of gene families [55]. Thus, we analyzed the duplication events of the *PHT* genes. The number of *PHTs* in the allotetraploid *B. napus* (108) is approximately 1.2-fold than in diploid *B. rapa* (54) plus *B. oleracea* (34). The number of *PHTs* in the allotetraploid *B. juncea* (85) is less than that in *B. rapa* (54) plus *B. nigra* (55) (Figure 2 and Table 1). These findings suggest that the *PHT* family evolved from diploid *B. rapa* and *B. oleracea* to tetraploid *B. napus* mainly through whole-genome duplication (WGD) but that it evolved from diploid *B. rapa* and *B. nigra* to tetraploid *B. juncea* mainly through gene loss. As shown in Figure 2, most homologous gene pairs between different genomes were contained in the corresponding localization blocks. For example, members of subgroups *PHT5:3*, *PHT3:1*, and *PHT4:5* were mapped to the tops of chromosomes A01 (BnaA01, BraA01, and BjuA01) and A02 (BnaA02, BraA02, and BjuA02), and *PHT4:2* and *PHT1:4* were localized to the bottom of chromosome A04 (BnaA04, BraA04, and BjuA04). Therefore, most *PHT* family members were localized to the corresponding positions of chromosomes with high levels of synteny among the five *Brassica* species (Figure 2). In addition, few *PHTs* were mapped onto chromosome BjuA05 (1), BjuB03 (2), and BjuB07 (1), and there were fewer *PHTs* in the allotetraploid species than in diploid species *B. rapa* and *B. nigra* (Figure 2), suggesting that they might have lost *PHT* genes after the polyploidization events.

Gene clusters (included two or more genes) within a 100-kb range of the same chromosomal regions were defined as tandem duplication [55]. We identified 114 *PHT* family members that were clustered into 40 tandem duplication event regions in the A, B, and C subgenomes from *B. rapa* (8), *B. oleracea* (5), *B. nigra* (5), *B. juncea* (10), and *B. napus* (12) (Figure 2). However, different orthologous copies of most *PHT1* gene(s) were retained in the tandem duplication regions, such as *PHT1:4* in chromosomes BnaA05, BraA05, and BniB04 and *PHT1:9* in chromosomes BnaA07, BraA07, BjuA07, and BniB07 (Figure 2).

**Figure 3:**
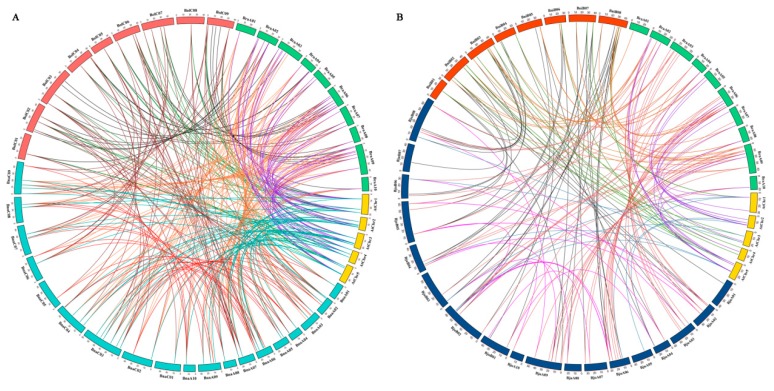
Genome-wide synteny analysis of *PHT* family genes among *Arabidopsis* and five *Brassica* species. (**A**) Collinearity analysis of *PHT* family genes among Arabidopsis, *B. napus, B. rapa,* and *B. oleracea.* (**B**) Collinearity analysis of *PHT* family genes among Arabidopsis, *B. juncea, B. rapa, and B. nigra.* Five Arabidopsis chromosomes (AtChr1–5), 19 *B. napus* chromosomes (BnaA01-10 and BnaC01-09), 18 *B. juncea* chromosomes (BjuA01-10 and BjuB01-08), 10 *B. rapa* chromosomes (BraA01-10), 9 *B. oleracea* chromosomes (BolC01-09), and 8 *B. nigra* chromosomes (BniB01-08) are shown, which are represented by different colored bars. Different gene pairs are represented by different colored lines in the figure.

To explore the amplification mechanism of *PHT* family genes, we performed synteny analysis of *PHTs* among *A. thaliana* and the five *Brassica* species. Collinearity analysis indicated that most syntenic orthologous *PHT* family gene pairs among the five *Brassica* species were retained in collinear blocks in different subgenomes (Figure 3, Figure S1 and Table S2). We identified 101, 27, and 59 orthologous *PHT* genes between *B. rapa* and *B. napus*, *B. rapa* and *B. oleracea*, and *B. oleracea* and *B. napus* (Figure 3A and Figure S1A) and 60, 32, and 53 orthologous *PHT* genes between *B. rapa* and *B. juncea*, *B. rapa* and *B. nigra*, and *B. nigra* and *B. juncea*, respectively (Figure 3B and Figure S1B). In addition, 32 and 16 orthologous *PHT* genes were detected in the BnaA and BnaC subgenomes of *B. napus* and the BjuA and BjuB subgenomes of *B. juncea*, respectively. These results indicate that these *PHT* family genes have a high degree of retention among the five *Brassica* species or experienced less loss when the diploid species (*B. rapa, B. oleracea*, and *B. nigra*) hybridized with the allotetraploids (*B. juncea* and *B. napus*) on the corresponding chromosomes during evolution [56].

We estimated the nonsynonymous substitution rate (Ka), synonymous substitution rate (Ks), and Ka/Ks ratio for each pair of duplicated genes among *A. thaliana* and the five *Brassica* species. The Ka/Ks ratios of the duplicates were less than 1, except for BraA02g031250 and BjuO002713, BjuB020400 and BniB012386, BnaA09g16490D and BnaC09g17550D, and BraA03g039090 and BnaA03g35470D (Table S2). These results suggest that most of these *PHT* family genes were subjected to purifying selection after duplication.

### 2.4. Gene Structural and Conserved Motif Analyses of PHT Family Genes in B. napus

**Figure 4:**
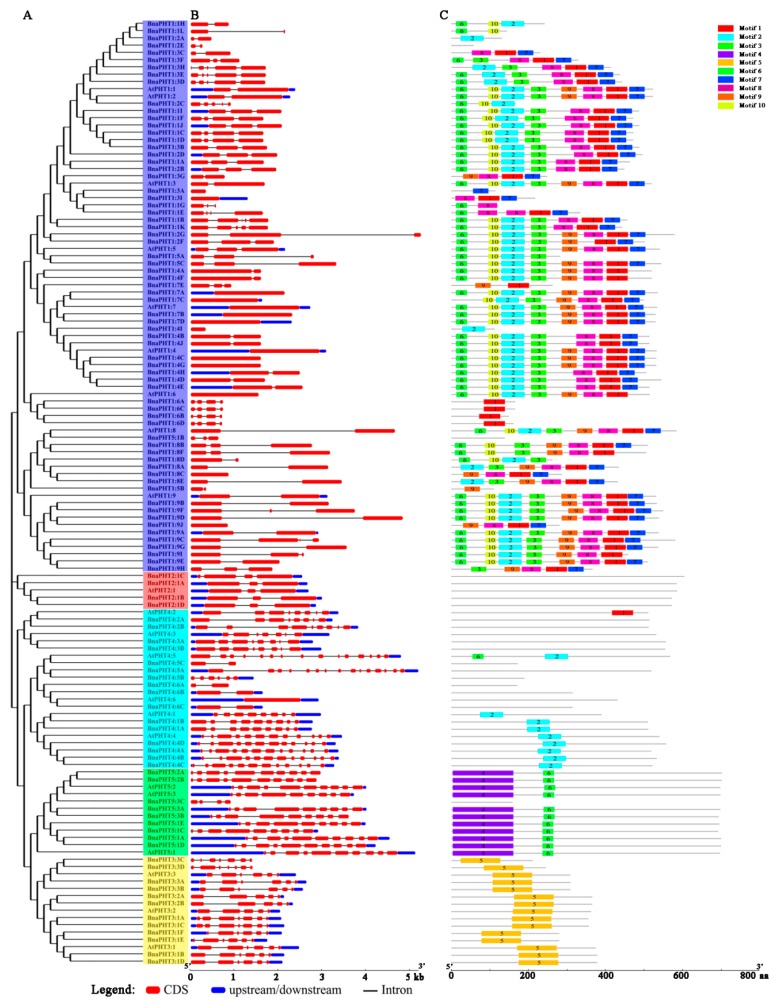
Analysis, gene structures, and protein motifs of *PHT* genes between *A. thaliana* and *B. napus*. (**A**) Phylogenetic tree. Full-length coding sequence (CDS) were aligned with Clustal X 2.0, and the phylogenetic tree was constructed using the neighbor-joining method. (**B**) Gene structures. Red boxes represent exons and gray lines represent introns. The untranslated regions (UTRs) are indicated by blue boxes. The sizes of the exons and introns can be estimated using the scale at the bottom. (**C**) Protein motifs. Conserved motifs (1–10) are represented by different colored boxes, whereas nonconserved sequences are indicated by gray lines.

To further explore the structural components of the *BnaPHT* genes, we analyzed the exon/intron arrangements of 22 *AtPHTs* and 108 *BnaPHTs* by comparing the full-length coding sequences (CDS) and the corresponding genomic DNA sequences using GSDS v2.0 (http://gsds.cbi.pku.edu.cn/index.php) based on phylogenetic analysis (Figure 4A). The 130 *PHTs* contained multiple numbers of exons varying from 1 to 15, indicating a high degree of divergence among genes (Figure 4B and Table S1). For example, members of the *PHT1* and *PHT2* subgroups contained fewer than five exons, whereas members of the *PHT3, PHT4*, and *PHT5* subgroups appeared to be more variable, with exon numbers ranging from 2 to 15 (Figure 4B). In general, the most closely grouped genes had highly similar structures in terms of intron numbers or exon lengths, and *BnaPHT* genes in different categories exhibited different exon/intron structural features.

Subsequently, we compared the distribution of conserved motifs to better explore the specific regions of the 22 AtPHT and 108 BnaPHT proteins. Ten distinct conserved motifs (named Motif 1–10) were captured by MEME v4.12.0 (http://meme-suite.org/tools/meme) (Figure 4C). The motifs ranged from 10 to 159 amino acids in size (Figure S2). Similar conserved motif compositions were shared among members of each group identified by phylogenetic analysis. Whereas variable motifs, including motif 1, 2, 3, 6, 7, 8, 9, and 10, were widely detected in the PHT1 proteins, and motifs 6 and 7 were comprised on the N and C-terminal domains of PHT1, respectively, and BnaPHT1:6 only contained motif 1. In addition, members of the PHT2 subgroup had no motifs, PHT3 subgroup members only contained motif 5, PHT4 subgroup members had different motif compositions (motif 2 in PHT4:1 and PHT4:4, others contained few motifs), and all PHT5 subgroup members contained motifs 4 (in N-terminal) and 6, except for BnaPHT5:3C (Figure 4C). In summary, the same conserved motifs are widely shared among paralogous/orthologous genes, suggesting they encode proteins with similar functions.

### 2.5. Transcriptional Patterns of BnaPHT Family Genes in B. napus

**Figure 5:**
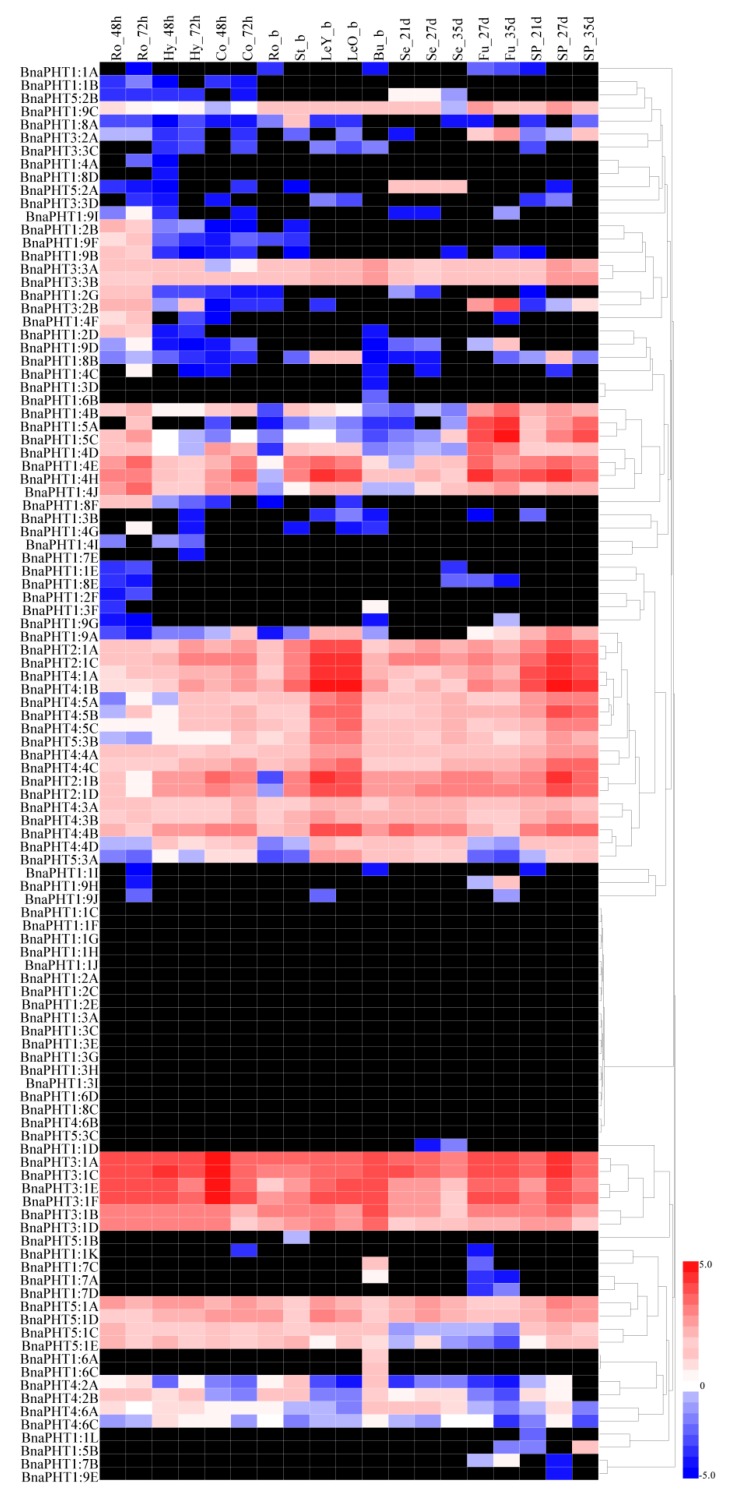
Heatmap of the expression profiles of *BnaPHT* family genes in different tissues and organs. The abbreviations above the heatmap indicate the different tissues and organs/developmental stages of *B. napus ZS11* (Table S3). The expression data were obtained from RNA-seq data and are shown as log2 values, as calculated based on FPKM values (fragments per kilobase of exon model per million). The heatmap was generated using Heatmap Illustrator 1.0 (HemI 1.0). The black indicates that the *BnaPHT* had no expression (FPKM = 0) levels in this study (Table S4).

Based on a transcriptional sequencing data set for *B. napus ZS11* (BioProject ID PRJNA358784), we characterized the expression profiles of *BnaPHT* genes in different tissues, including radicle, hypocotyl, cotyledon, root, stalk, young leaf, old leaf, bud, seed, funicle, and seed coat tissue (Table S3). Heat map analysis showed that most *BnaPHTs* were differentially expressed in different tissues at different stages of development in *B*. *napus* (Figure 5 and Table S4). For instance, most *BnaPHT1* subfamily members showed little or no detectable expression, but *BnaPHT2*, *BnaPHT3*, *BnaPHT4*, and *BnaPHT5* were consistently expressed at high levels in most tissues, suggesting that *BnaPHTs* are involved in multiple processes during plant growth and development. Most *BnaPHT1* genes showed almost no expression in any tissue, whereas most *BnaPHT1:4* and *BnaPHT1:5* genes were expressed at high levels during *B*. *napus* development (Figure 5 and Table S4). However, most members of subgroups *BnaPHT2* to *BnaPHT5* showed different expression patterns except for *BnaPHT4:6B, BnaPHT5:1B,* and *BnaPHT5:3C*. For instance, four *BnaPHT2:1* subgroup members (*BnaPHT2:1A-D*) and six *BnaPHT3:1* subgroup members (*BnaPHT3:1A to BnaPHT3:1F*) were expressed at relatively high levels throughout development, whereas *BnaPHT4* subgroup members were primarily expressed in leaves (Figure 5). The expression patterns of *BnaPHT* family genes also corresponded with the results of phylogenetic analysis (Figure 1). For example, the expression patterns of *BnaPHT3:1E* and *BnaPHT3:1F*, *BnaPHT3:1A* and *BnaPHT3:1C,* and *BnaPHT4:1A* and *BnaPHT4:1B* were similar. These genes were classified into the same sister groups and are syntenic genes (Figure 1 and Figure 5). These results demonstrate that most *BnaPHTs* have tissue-specific or preferential expression patterns.

### 2.6. BnaPHT Transcript Patterns in Response to As^3+^ and Cd^2+^ Treatment

**Figure 6:**
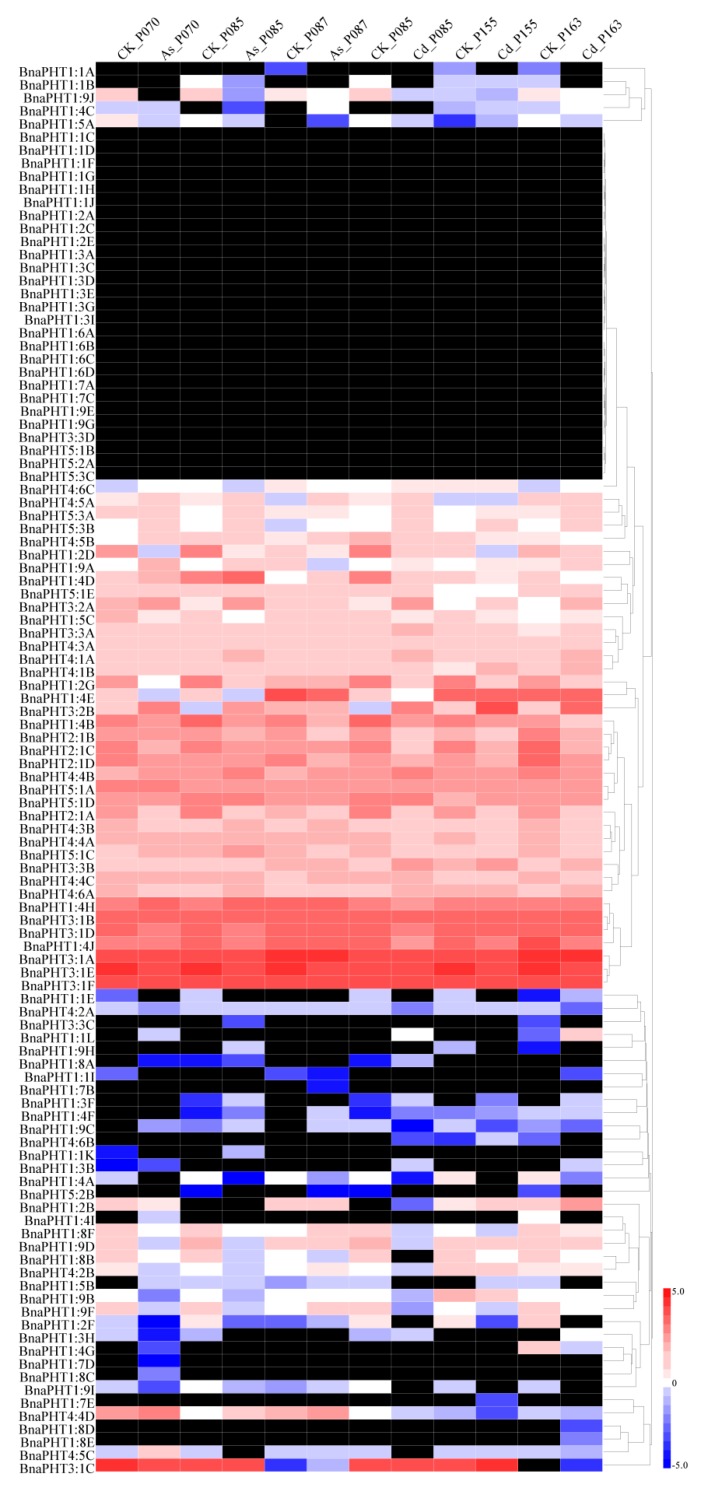
Heatmap of the expression profiles of *BnaPHT* family genes in different rapeseed accessions treated with As^3+^ and Cd^2+^. The samples and treatments are shown above the heatmap. The results were obtained by RNA-seq analysis. The relative expression values in the bars were calculated based on FPKM values (fragments per kilobase of exon model per million) compared to the control samples. The heatmap was generated using Heatmap Illustrator 1.0 (HemI 1.0). The black indicates that the *BnaPHT* had no expression (FPKM = 0) levels in this study (Table S5).

As PHT family proteins also function in the acquisition of heavy metals [46,49,50,51], we next selected rapeseed accessions with different levels of resistance to As^3+^ (3) and Cd^2+^ (3) and subjected them to 15 mg/L As^3+^ and 30 mg/L Cd^2+^ treatment (Figure S3). Subsequently, we compared the expression levels of *PHT* genes in the rapeseed accessions under As^3+^ and Cd^2+^ treatment using a recently developed RNA-seq method. Most *BnaPHT* genes were not obviously induced by either heavy metal (As^3+^ or Cd^2+^), including *BnaPHT1:1*, *BnaPHT1:3*, *BnaPHT1:6,* and *BnaPHT1:7*, while a few *PHT* family members were up- or downregulated by these treatments. For example, *BnaPHT1:4A*, *BnaPHT1:4B*, *BnaPHT1:4E*, *BnaPHT2:1,* and *BnaPHT4:2* were downregulated by treatment with both heavy metals (As^3+^ and Cd^2+^), and *BnaPHT1:9A*, *BnaPHT3:2*, *BnaPHT4:5A*, *BnaPHT5:1D*, *BnaPHT5:3A,* and *BnaPHT5:3B* were upregulated by both treatments (Figure 6 and Table S5). In addition, a few *PHT* family members showed different expression patterns after treatment with As^3+^ vs. Cd^2+^. For instance, *BnaPHT1:4D*, *BnaPHT1:4H*, *BnaPHT1:9C,* and *BnaPHT4:5B* were upregulated by As^3+^ treatment but downregulated by Cd^2+^ treatment, and *BnaPHT3:3A* and *BnaPHT3:3B* showed the reverse expression pattern (Figure 6 and Table S5). *PHT* family members including *BnaPHT1:2B*, *BnaPHT1:8F*, *BnaPHT4:4A*, *BnaPHT4:4C*, *BnaPHT4:5C*, and *BnaPHT5:1C* were repressed by Cd^2+^ but not As^3+^ treatment (Figure 6 and Table S5).

In summary, our results indicate that *BnaPHT* family genes have a wide variety of expression patterns in *B. napus*, and a few *BnaPHTs* are obviously induced by heavy metals, providing useful information for further elucidating the roles of *BnaPHTs* in the response of *B. napus* to heavy metal stress.

### 2.7. Expression Analysis of BnaPHTs under As^3+^ and Cd^2+^ Treatment 

**Figure 7:**
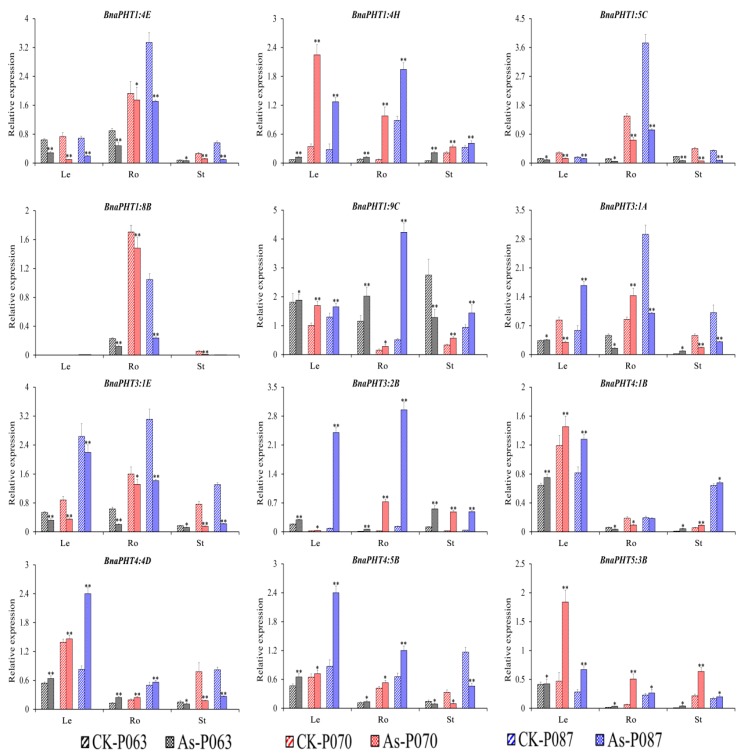
Expression profiles of 12 *BnaPHT* genes in *B*. *napus* under As^3+^ treatment, as revealed by qRT-PCR. The CK is the control sample, and As represents samples treated with As^3+^ stress. The samples (P063, P070, and P087) are *B*. *napus* accessions. Error bars show the standard deviation of three biological replicates. Single and double asterisks represent significant differences from the control sample at the 0.05 and 0.01 levels (t-test), respectively.

**Figure 8:**
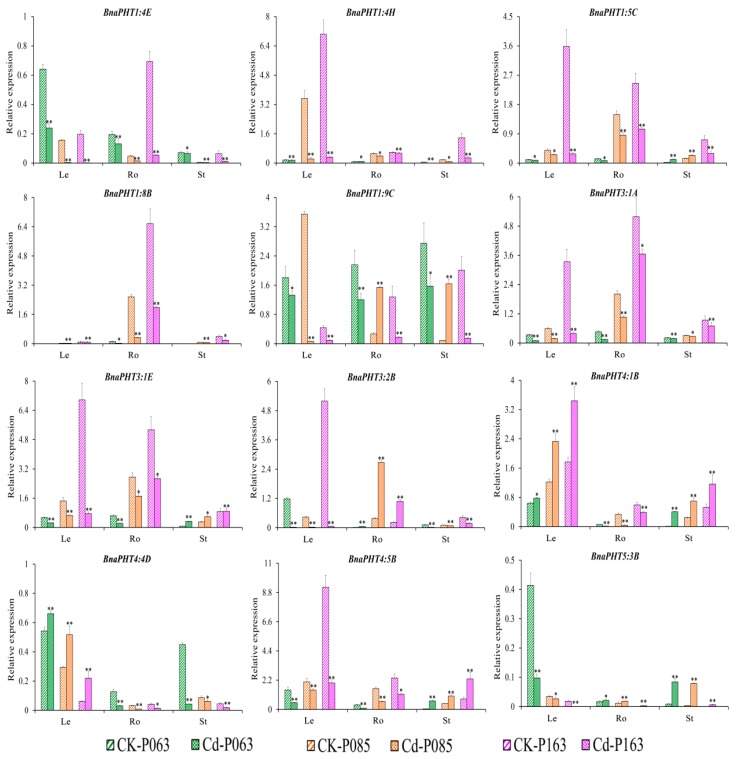
Expression profiles of 12 *BnaPHT* genes in *B*. *napus* under Cd^2+^ treatment, as revealed by qRT-PCR. The CK is the control sample, and Cd represents samples treated with Cd^2+^ stress. The samples (P063, P085, and P0163) are *B*. *napus* accessions. Error bars show the standard deviation of three biological replicates. Single and double asterisks represent significant differences from the control sample at the 0.05 and 0.01 levels (t-test), respectively.

To confirm the role of *BnaPHTs* in plant tolerance to heavy metal stress, we subjected various rapeseed accessions to one week of heavy metal treatment (As^3+^ or Cd^2+^). Different accessions showed different responses to these treatments (Figure S3). Based on the transcriptome data, we selected 16 genes of interest for further verification by qRT-PCR (Table S6). The expression patterns of the *BnaPHTs* identified by qRT-PCR were similar to the patterns identified by RNA-seq (Table S5), with different expression profiles detected among different rapeseed accessions (Figure 7 and Figure 8). The expression profiles of four genes (*BnaPHT1:1C*, *BnaPHT1:7C*, *BnaPHT3:3D,* and *BnaPHT5:1B*) are not shown because they were expressed at very low levels in all organs under As^3+^ and Cd^2+^ treatment.

Under As^3+^ treatment, four *BnaPHTs* (*BnaPHT1:4E*, *BnaPHT1:5C*, *BnaPHT1:8B,* and *BnaPHT3:1E*) were downregulated and three *BnaPHTs* (*BnaPHT1:4H*, *BnaPHT3:2B,* and *BnaPHT5:3B*) were upregulated in different organs (roots, hypocotyls, and cotyledons), with similar trends in expression. However, the relative expression levels of these genes significantly differed among samples and tissues (Figure 7), suggesting they represent important candidate genes that function in As^3+^ stress resistance in *B. napus*. Under As^3+^ treatment, *BnaPHT1:9C*, *BnaPHT4:4D,* and *BnaPHT4:5B* were significantly induced in leaves and roots, whereas *BnaPHT4:4D* and *BnaPHT4:5B* were significantly repressed in stems. *BnaPHT4:1B* was significantly induced in leaves and stems but repressed in roots in response to As^3+^ treatment (Figure 7). These results suggest that these genes have different functions in different organs.

Under Cd^2+^ treatment, various genes (*BnaPHT1:4E*, *BnaPHT1:4H*, *BnaPHT1:8B,* and *BnaPHT3:1A*) were significantly downregulated in different organs (roots, hypocotyls, and cotyledons). By contrast, *BnaPHT4:1B* was upregulated in leaves and stems but downregulated in roots under Cd^2+^ treatment, while *BnaPHT5:3B* was significantly upregulated in roots and stems but downregulated in leaves (Figure 8). In addition, *BnaPHT3:1E, BnaPHT3:2B,* and *BnaPHT4:4D* were upregulated in stems, roots, and leaves but downregulated in other organs in response to Cd^2+^ treatment (Figure 8). These results suggest that these genes have different functions in the plant response to Cd^2+^ stress.

In summary, several genes (*BnaPHT1:4E*, *BnaPHT1:8B,* and *BnaPHT4:1B*) showed similar expression patterns in response to As^3+^ and Cd^2+^ stress, suggesting they might function in heavy metal tolerance. However, other genes were also induced by As^3+^ or Cd^2+^ stress to a certain extent but showed different expression patterns in different organs. Therefore, the specific mechanism that regulates the expression of the genes that affect heavy metal stress tolerance in rapeseed requires further investigation.

## 3. Discussion

Plants of the genus *Brassica* are widely used as oilseed and vegetable crops and to produce condiments and animal feed worldwide. The major *Brassica* crops include three diploid species (*B. rapa*, *B. nigra*, and *B. oleracea*) and three allotetraploid species (*B. napus*, *B. juncea,* and *B. carinata*) [57,58,59]. With the completion of the genome sequences of these *Brassica* species, the genetic and molecular functions of interesting genes, such as AP2/ERF, MYB, BOR, CO-like, and GRAS genes, have been widely studied in *B. rapa* and *B. napus* [60,61,62,63,64,65]. *PHT* genes encode H_2_PO^4^**^−^**/H^+^ co-transporters that are responsible for the absorption and transport of phosphorus in plant roots. The functions of *PHT* family members have been studied in Arabidopsis, rice, populous, and soybean (*Glycine max*) [1,66,67], but few studies have focused on *PHT* genes in *Brassica* species.

The *PHT1* family in Arabidopsis has nine members, eight of which are expressed in roots (e.g., *AtPHT1;1*, *AtPHT1;4*, *AtPHT1;8,* and *AtPHT1;9)* and play crucial roles in Pi acquisition in both low- and high-P environments. In rice (*Oryza sativa*), the *PHT1* family has 13 members, including *OsPHT1;2*, *OsPHT1;6*, *OsPHT1;1*, and *OsPHT1;8*, which play redundant roles in root-to-shoot Pi translocation. The *PHT1* family has also been studied in *Medicago truncatula*. *MtPT1;1*, *MtPT1;2*, *MtPT1;3* and *MtPT1;5* are highly expressed in roots under Pi deprivation, but at lower levels in response to high Pi levels [14]. In addition, 49 *PHT1* family members were identified in *B*. *napus*. These genes might be involved in maintaining the external status of P, N, K, S, and Fe, as well as responses to salt and drought stress [4], but only *BnaPHT1:4* was demonstrated to be preferentially expressed in cotyledons during early seedling development [68]. Whereas few reports were focused on the *PHT3, PHT4,* and *PHT5* family genes, and *PHT3* family was first reported after cloning the plant mitochondrial transporter genes, which were highly conserved in the mitochondrial transporter family, *PHT4* family was similarly expressed in both mutant and wild-type plants, and *PHT4:1* may affect salicylic acid-mediated defense. Its expression was regulated by the circadian clock, whereas *PHT5:1* played an important role in the adaptation of plants to climatic fluctuation [26,32,69,70,71]. Recently, the entire *PHT* family (*PHT1, PHT2, PHT3, PHT4,* and *PHT5*) was identified from different plants [1,5,71]. In the present study, we conducted comprehensive analysis and identified 336 putative *PHT* family genes in *B*. *rapa* (54), *B*. *oleracea* (34), *B*. *nigra* (55), *B*. *juncea* (85), and *B. napus* (108). These genes were classified into five subgroups (*PHT1, PHT2, PHT3, PHT4*, and *PHT5*; Figure 1 and Table S1) based on the *PHT* subfamilies in *A. thaliana*, indicating that they share similar functions. We identified 201 *PHT1s*, including 34 in *B*. *rapa*, 14 in *B*. *oleracea*, 35 in *B*. *nigra*, 52 in *B*. *juncea,* and 66 in *B. napus*, indicating that this subfamily is larger in *Brassica* species than in others plants, such as Arabidopsis (9), barley (8), rice (13), populus (14), and tomato (8) [1,66,72,73,74]. In addition, we also identified 15 *PHT2s*, 40 *PHT3s*, 54 *PHT4s*, and 26 *PHT5s* in the five *Brassica* species. The number of members of each subgroup ranged from 2 to 4 for *PHT2*, 5 to 12 for *PHT3*, 7 to 16 for *PHT4,* and 4 to 10 for *PHT5* (Table S1). These results indicate that the *PHT* subgroups contain different numbers of members in different species and that they are larger in *Brassica* species than in Arabidopsis [2,21,26] and poplar [1]. The *PHT1* and *PHT2* subgroups contain 201 and 15 members, making them the largest and smallest groups in *Brassica*, respectively, suggesting that they play universal and unique roles in plants [75]. Interestingly, the numbers of *PHTs* were larger in this study than in previous studies except for *PHT2* family genes, such as 49 and 45 *PHT1* family genes in *B*. *napus* [4,5], while 66 in this study. The number of *PHT2* was consistent with the published results [5]. The results indicate that some *PHTs* were further identified in the future.

Polyploidy is widespread in plants and has been identified in more than 90% of flowering plants [76]. The allotetraploid *Brassica* species (*B. juncea* and *B. napus*) have experienced many duplication events after hybridizing with their diploid progenitors (*B*. *rapa*, *B*. *oleracea,* and *B*. *nigra*), and their gene numbers have increased during evolution, making them ideal plants for studying the role of polyploidization in evolution [76,77,78]. We determined that some *PHT* subfamilies were either larger or smaller in the allotetraploid species (*B. juncea* and *B. napus*) than the combined numbers in their corresponding diploid progenitors (*B*. *rapa*, *B*. *oleracea,* and *B*. *nigra*), suggesting that gene expansion and loss occurred in the *PHT* family in *Brassica* during the process of polyploidization, and showed the similar patterns with the published results [5]. A comparison of the genomes of the *Brassica* species (BraA, BolC, BniB, BjuA, BjuB, BnaA, and BnaC) revealed that the chromosomal distributions and gene numbers were consistent in the corresponding localization blocks between the diploid ancestral species and allotetraploid species, including members of subgroups *PHT5:3*, *PHT3:1, PHT4:5*, *PHT4:2,* and *PHT1:4* (Figure 2). Furthermore, we detected 40 tandem duplications (five in *B. oleracea*, eight in *B. rapa*, five in *B*. *nigra*, 10 in *B*. *juncea,* and 12 in *B. napus*) among the five *Brassica* species, including 114 *PHTs*, suggesting that these *PHT* family genes are functionally redundant. Additionally, our results indicate that purifying selection played a primary role in *PHT* family gene expansion among the five *Brassica* species, suggesting that these duplicate genes might still retain their ancestral functions.

Rapeseed is a good winter fallow crop and an excellent crop for remediating heavy metal pollution in soil due to its rapid growth, high biomass, and strong heavy metal tolerance due to its ability to absorb and accumulate these toxins [44,45]. Therefore, we further characterized the *BnaPHT* family genes, including their gene structures, motifs, localization patterns, and expression patterns (Figure 4, Figure 5, Figure 6, Figure 7 and Figure 8 and Table S2). *BnaPHTs* and *AtPHTs* within the same subgroups share similar gene structures, motifs, and localization patterns (Figure 4). In general, most *BnaPHT1* subgroup members share the fewest exons with multiple motifs. Most *BnaPHT2* subgroup members contain three exons but no conserved motifs. The *PHT3, PHT4*, and *PHT5* subgroups have more variable numbers of exons but contain specific motifs, such as motif 5 in *PHT3* and motif 4 in *PHT5* (Figure 4). Therefore, our classification and evolutionary analysis of *PHT* genes in *B. napus* suggested that *PHTs* within the same subgroup share relatively conserved roles [5], providing guidance for subsequent functional research.

The syntenic genes were highly conserved in terms of both their structures and expression patterns. A structural comparison of syntenic genes highlighted several similarities between *PHT* family genes among the five *Brassica* species and *A. thaliana* (Figure 2 and Figure 3 and Figure S1). To investigate the functions of these *PHT* genes in more detail, we examined their transcriptional patterns, which can provide important clues about their functions. We constructed a heatmap based on the relative expression levels of *PHT* genes in different *B. napus ZS11* tissues, including radicle, hypocotyl, cotyledon, root, stalk, young leaf, old leaf, bud, seed, funicle, and seed coat tissue (Figure 5). In general, homologous *PHT* family genes had different expression patterns in different tissues [1]. For instance, most *BnaPHT1* family members were not expressed in any tissue examined, but 14 *PHT1* family genes showed high expression levels in roots, in consistent with the function that they might involve in Pi uptake and translocation [3,5,15,18]. In addition, *BnaPHT1:4* showed the higher levels in developmental seeds, which also play an important role involved in the embryo development [5,79]. However, four *BnaPHT2:1* family members were highly expressed in leaves (Figure 5), and the homologs of these genes are predominantly expressed in green tissues in *Arabidopsis* [19] and rapeseed [5]. Our results supported the facts that functions of *PHT2s* are in the plastid inner envelope [19]. Recently, the genome-wide identification and analysis of the whole *PHT* family genes have been carried out in many plants [1,5,71], and other *PHT* family genes (*PHT3* to 5) were also identified, but few genes of them were still functionally characterized in plants. Here, we found that several gene pairs (*BnaPHT3:1E* and *BnaPHT3:1F*, *BnaPHT3:1A* and *BnaPHT3:1C*) have similar expression patterns, and are especially expressed in germinating seed tissues, highlighting the structural and functional similarity of syntenic genes and laying the foundation for complementation experiments to verify their functions. In addition, *BnaPHT5:3A* is located in the BnaAn genome and is syntenic to *BraPHT5:3A*, and *BnaPHT5:3A* is located in the BnaCn genome and is syntenic to *BolPHT5:3A* (Figure 2 and Figure 3). These genes belong to the PHOSPHATE TRANSPORTER 5 family (*PHT5*, also known as vacuolar phosphate transporter (VPT)), encoding vacuolar Pi transporters [70], which showed high expression levels in leaf and siliques (Figure 5). However, there were also cases in which there were large differences in expression levels between gene pairs. For example, *BnaPHT3:1F* was highly expressed in cotyledons, but *BnaPHT3:1D* was expressed at very low levels in this tissue (Figure 5). Perhaps the loss of genetic information during evolution resulted in structural changes to genes in these pairs, resulting in different protein functions. In addition, the results showed that *BnaPHT4s* were relatively divergent [5], but we found most *BnaPHT4s* had higher expression levels in leaf and siliques (Figure 5), which were mostly located in the plastid envelope and one Golgi-localized transporter basal defense [19]. Furthermore, *BnaPHT4:4* had higher expression profiles in leaf and siliques, which are in agreement with their biological roles in a chloroplast-localized ascorbate transporter [29]. Similarly, we also found *BnaPHT5:1* had the stable expression levels, supporting the facts that *PHT5* genes are required for fitness and plant growth [70]. In brief, our results suggest that *PHT* genes have different functions, since they are expressed in a wide range of tissues in *B*. *napus*.

In addition, *PHT* family genes are specifically expressed in various tissues in response to heavy metal stress [46,49,50,51]. Therefore, we analyzed the expression patterns of *BnaPHT* genes in response to As^3+^ and Cd^2+^ treatment by RNA-seq analysis. The expression patterns of *BnaPHT* family genes differed under As^3+^ and Cd^2+^ induction. For example, while *BnaPHT1:1*, *BnaPHT1:3*, *BnaPHT1:6,* and *BnaPHT1:7* were not induced in rapeseed in response to As^3+^ or Cd^2+^ treatment, *BnaPHT1:4A, BnaPHT1:4B, BnaPHT1:4E, BnaPHT2:1,* and *BnaPHT4:2* were downregulated and *BnaPHT1:9A*, *BnaPHT3:2*, *BnaPHT4:5A*, *BnaPHT5:1D*, *BnaPHT5:3A,* and *BnaPHT5:3B* were upregulated by these treatments (Figure 6). These results were further confirmed by qRT-PCR analysis, pointing to the reproducibility and reliability of our results. For example, *BnaPHT1:4E*, *BnaPHT1:8B,* and *BnaPHT4:1B* shared similar expression patterns in different organs under As^3+^ and Cd^2+^ treatment, whereas most *BnaPHTs* showed different expression patterns (Figure 7 and Figure 8), suggesting they play different roles in different stress responses. This study represents the first comprehensive analysis of *BnaPHT* family genes under heavy metal stress, laying the foundation for further elucidating the roles of *PHT* genes in heavy metal tolerance.

## 4. Materials and Methods 

### 4.1. Identification of PHT Family Genes in Brassica

The amino acid sequences of the *PHTs* were downloaded from The Arabidopsis Information Resource (TAIR10) database (ftp://ftp.arabidopsis.org), which was used as queries for protein basic local alignment search tool (BLASTp) [80] analysis against whole-genome sequences in the *Brassica* database. The hidden Markov model (HMM) search program (HMMER v3.0, http://hmmer.janelia.org/) was used to identify and validate candidate sequences with E-value ≤ 1 × 10^−20^. BLAST analysis of the *PHTs* was performed against a *Brassica* protein database constructed using Geneious v4.8.5 software (http://www.geneious.com/, Biomatters, Auckland, New Zealand). The coding sequences (CDS) of the *PHTs* were identified by BLASTn searches against the *Brassica* genome database. The candidate proteins were named using the species abbreviation of the source organism (italicized), the gene family name, and the positions in the subtribe, e.g., *AtPHT1;1* and *BnaPHT1;1A*. The physicochemical properties of the predicted PHT proteins, such as isoelectric point (pI), MW, instability index, and grand average of hydropathicity (GRAVY), were analyzed using the ProtParam tool of ExPASy server [80] (https://web.expasy.org/protparam/).

### 4.2. Multiple Sequence Alignment and Phylogenetic Analysis of PHTs in Brassica

The deduced amino acid sequences of PHT proteins from *A. thaliana* and various *Brassica* species, including *B. rapa*, *B. oleracea*, *B. napus*, *B. juncea*, and *B. nigra,* were subjected to multiple protein sequence alignment using ClustalW software [81] with default settings. To illustrate the evolutionary relationships of the *PHTs* in *Brassica*, a neighbor-joining [27] phylogenetic tree was generated using the MEGA v7.0 program (Tokyo Metropolitan University, Tokyo, Japan) [82] with the Jones-Taylor-Thornton (JTT) + invariant sites (I) + Gamma (G) substitution model and a bootstrap test with 1000 replicates. The phylogenetic trees were visualized using FigTree v1.4.2 (http://tree.bio.ed.ac.uk/software/figtree/).

### 4.3. Conserved Motif Recognition and Gene Structure Analysis

The CDS of the *PHTs* from the five *Brassica* species were retrieved based on their protein sequences, and the corresponding genomic sequences were extracted from the *Brassica* genome sequences. The exon-intron structures of the *PHTs* were analyzed online using the Gene Structure Display Server (GSDS v2.0, http://gsds.cbi.pku.edu.cn/index.php). Conserved motifs were identified using Multiple Expectation Maximization for Motif Elucidation (MEME v4.12.0, http://meme-suite.org/tools/meme) [83] with the following parameters: Number of repetitions, any; maximum number of motifs, 10; and optimum width of each motif, between 6 and 300 residues. Each motif with an *E*-value < 1 × 10^−10^ was retained for motif detection.

### 4.4. Chromosomal Locations of PHT Family Genes

The *PHT* family genes were mapped to the *Brassica* chromosomes based on their physical distances in the general feature format (GFF) genome files, which were downloaded from the *Brassica* database (BRAD, http://brassicadb.org). Then the MapChart v2.0 [84] was used to construct the chromosome localization map of the *PHTs*.

### 4.5. Analysis of Duplication and Synteny of PHT Family Genes in A. thaliana and Five Brassica Species

Ancient duplication events were investigated to confirm the gene duplication events. The nonsynonymous substitution rate (Ka), synonymous substitution rate (Ks), and Ka/Ks ratio for each pair of duplicated genes among *A. thaliana* and the five *Brassica* species were computed between pairs of genes identified as homologous using TBtools_JRE1.6 with default settings [85]. To explore the amplification mechanism of *PHT* family genes, we performed synteny analysis of *PHTs* among *A. thaliana* and the five *Brassica* species. We used information such as chromosome length, gene location, and homologous gene information derived from TBtools and used TBtools-Multiple Synteny Plotter with default parameters to perform synteny detection. Simultaneously, TBtools-Amazing Super Circos was used to illustrate the homologous genetic relationships between different species.

### 4.6. Plant Materials and Metal Stress Treatments

*B. napus* seeds were obtained from the Rapeseed Engineering Research Center of Southwest University in Chongqing, China (CERCR). Then we performed a trial to identify the optimal concentration of as and Cd treatment in *B*. *napus*. Moreover, the length of root and radicle of five randomly selected rapeseed showed wider variation under 15 mg/L As and 30 mg/L Cd treatment than in other concentrations (0, 5, 10, 15, and 20 mg/L As, and 0, 10, 20, 30, and 40 mg/L Cd). Thus, 15 mg/L As and 30 mg/L Cd were used as a most optimal concentration in this study. Subsequently, 200 *B. napus* accessions were treated with different concentrations of two heavy metals (As^3+^ and Cd^2+^), and 14 accessions with extreme phenotypes (strong or weak resistance) were obtained based on phenotypic differences in their growth. 

The healthy seeds (0.3 g) were selected from each accession and sown on four layers of filter paper in the Petri dishes (d = 9 cm) with distilled water (the control) and 15 mg/L As or 30 mg/L Cd. Seeds were generated at 25 °C with long-day conditions (16 h light/8 h dark, 5000 Lux). After 7 days, whole roots, hypocotyls, and cotyledons were sampled to analyze *PHT* gene expression patterns. The tissues were snap frozen in liquid nitrogen and stored at −80 °C prior to total RNA extraction.

### 4.7. Total RNA Extraction and qRT-PCR Analysis

The growth of *B. napus* accessions P063, P070, and P087 was significantly inhibited under treatment with the heavy metal As, and the growth of P063, P085, and P163 was significantly inhibited under Cd treatment. These accessions were, therefore, used for expression analysis. Total RNA was isolated from the samples using a DNAaway RNA Mini-prep Kit (Sangon Biotech, Shanghai, China). For tissue-specific expression analysis, RNA was extracted from roots, hypocotyls, and cotyledons and pretreated with genomic DNA (gDNA) Eraser (Takara, Dalian, China). Subsequently, 1 μg total RNA was used to synthesize first-strand complementary DNA (cDNA) with an RNA PCR Kit (AMV) Ver. 3.0 (Takara, Dalian, China). The cDNA was subjected to qRT-PCR analysis using SYBR Premix Ex Taq II (Takara, Dalian, China) on a Bio-Rad CFX96 Real Time System (Bio-Rad Laboratories, Hercules, CA, USA) as previously described [86]. *BnACTIN7* (EV116054) was employed as a reference gene to normalize *PHT* gene expression levels via the 2^−∆∆*C*t^ method [87]. All experiments were performed with three technical replicates, and the values represent the average + standard error (SE). One-way ANOVA (** *p* < 0.01, * *p* < 0.05) was used to determine the significance level of the data using Excel software. The specific primer sequences used in this study were obtained from the qPCR Primer Database [88] and are listed in Table S6.

## 5. Conclusions

In the present study, we identified 336 *PHT* genes from five *Brassica* species and divided them into five subgroups: *PHT1, PHT2, PHT3, PHT4*, and *PHT5*. We performed a comprehensive bioinformatics analysis, including an analysis of the chromophore position, gene structure, conserved domain structure, and evolutionary relationships of the genes, to characterize *PHT* family genes in the five species. Our analysis suggests that segmental and tandem duplications and gene loss events occurred during the expansion of the *PHT* gene family during the process of polyploidization. Moreover, purifying selection played a major role in the expansion of *PHT* family genes among the five *Brassica* species. Finally, we explored the expression profiles of the *BnaPHT* family genes in specific tissues, at various developmental stages, and in response to heavy metal stress via RNA-seq analysis and qRT-PCR. The results shed light on the evolution of *PHT* family genes and provide a reference for future polyploidization analysis. Our findings provide a basis for further studies of the roles of *BnaPHTs* in plant tolerance to heavy metal stress.

## Figures and Tables

**Table 1:** Statistics of *PHT* genes in each *PHT* subroup between *Arabidopsis thaliana* and five *Brassica* species.

Type	Family	*A. thaliana*	*B. rapa*	*B. oleracea*	*B. nigra*	*B. juncea*	*B. napus*
*PHT1*	*PHT1:1*	1	3	2	4	3	12
	*PHT1:2*	1	4	1	7	4	7
	*PHT1:3*	1	5	/	3	6	9
	*PHT1:4*	1	5	1	5	10	10
	*PHT1:5*	1	1	/	2	2	3
	*PHT1:6*	1	2	2	3	6	4
	*PHT1:7*	1	3	/	2	5	5
	*PHT1:8*	1	2	3	3	5	6
	*PHT1:9*	1	8	5	7	11	10
*PHT2*	*PHT2:1*	1	2	3	2	4	4
*PHT3*	*PHT3:1*	1	3	2	3	6	6
	*PHT3:2*	1	1	2	1	2	2
	*PHT3:3*	1	2	2	1	3	4
*PHT4*	*PHT4:1*	1	1	1	2	2	2
	*PHT4:2*	1	1	1	2	2	2
	*PHT4:3*	1	1	1	1	2	2
	*PHT4:4*	1	2	2	2	3	4
	*PHT4:5*	1	1	1	1	2	3
	*PHT4:6*	1	2	1	1	3	3
*PHT5*	*PHT5:1*	1	2	2	2	2	5
	*PHT5:2*	1	1	1	1	1	2
	*PHT5:3*	1	1	1	1	1	3

## Supplementary Materials

Supplementary materials can be found at https://www.mdpi.com/1422-0067/21/6/2209/s1. Table S1. List of PHT genes identified in the *A. thaliana* and *Brassica* genomes; Table S2. One-to-one orthologous relationships between A. thaliana and other five plant species; Table S3. *B. napus* ZS11 tissues and organs used in this study; Table S4. The FPKM values of PHT family genes in *B*. *napus* by RNA-Seq analysis; Table S5. The relative expression values of PHT family genes in *B. napus* by RNA-Seq analysis under As^3+^ and Cd^2+^ treatment; Table S6. Primers used to amplify the PHT genes and reference genes using qRT-PCR; Figure S1. Collinearity analysis of *PHT* family genes among *Arabidopsis* and five *Brassica* species; Figure S2. The detailed information of motif logos are obtained from the MEME Suite website; Figure S3. The phenotypes of rapeseed under As^3+^ and Cd^2+^ stress.
