# Supplementary material for: Genome-Wide Analysis of Phosphorus Transporter Genes in Brassica and Their Roles in Heavy Metal Stress Tolerance
Source: Int J Mol Sci. 2020 Mar 23;21(6):2209. doi: 10.3390/ijms21062209 (PMC7139346; doi:10.3390/ijms21062209)
Supplement: Supplementary file 1 [file ijms-21-02209-s001.zip › supplementary figures.docx]

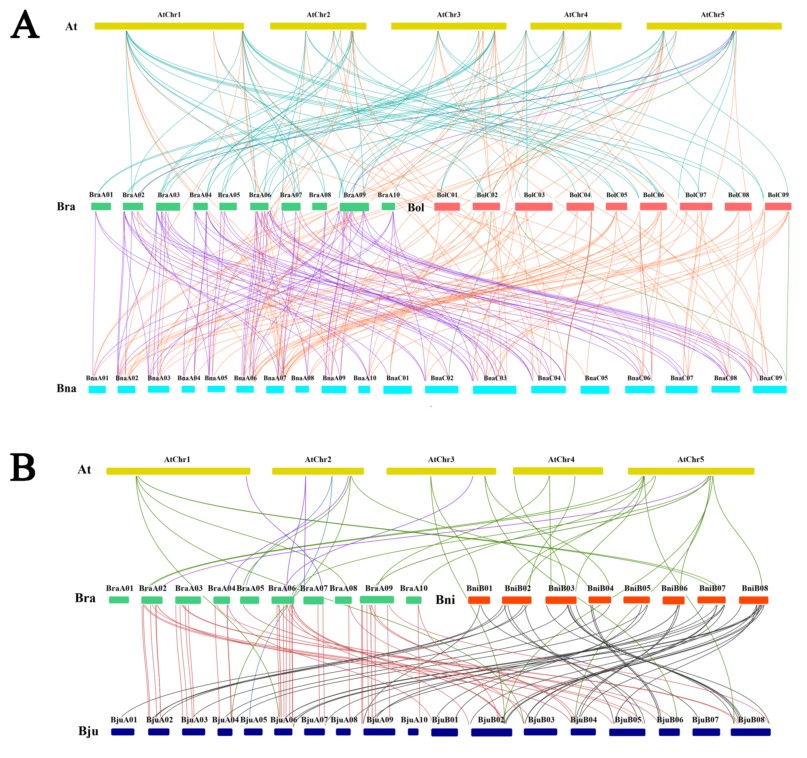


**Figure S1.** Collinearity analysis of *PHT* family genes among *Arabidopsis* and five *Brassica* species.


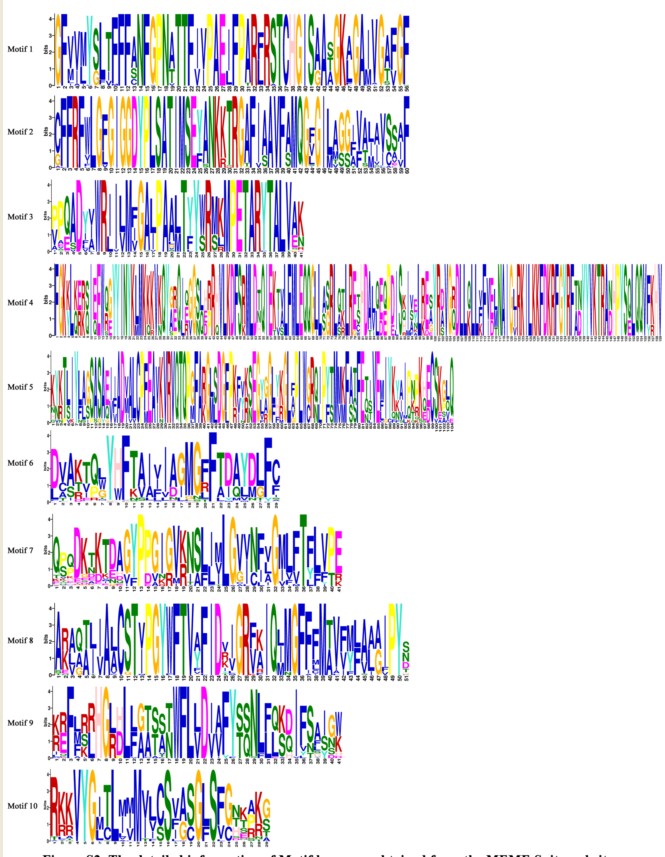


**Figure S2.** The detailed information of motif logos are obtained from the MEME Suite website.


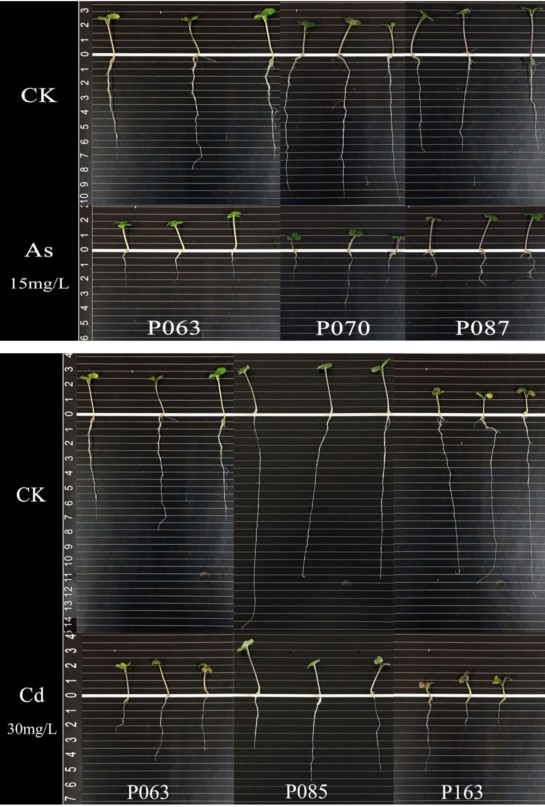


**Figure S3.** The phenotypes of rapeseed under As^3+^ and Cd^2+^ stress.
